# Polydopamine-Based Composite Nanoparticles with Redox-Labile Polymer Shells for Controlled Drug Release and Enhanced Chemo-Photothermal Therapy

**DOI:** 10.1186/s11671-019-3027-6

**Published:** 2019-05-30

**Authors:** Yefei Tian, Miao Lei

**Affiliations:** 1 School of Materials Science and Engineering, Chang’an University, Xi’an, 710064 Shaanxi People’s Republic of China; 2 Engineering Research Central of Pavement Materials, Ministry of Education of PR China, Chang’an University, Xi’an, 710064 People’s Republic of China

**Keywords:** Disulfide bond, Redox-responsive, Controlled drug release, Chemo-photothermal therapy

## Abstract

Photothermal therapy (PTT) that utilizes phSUPPotothermal conversion agents (PTC) to ablate tumor under NIR light irradiation has attracted increasing attention due to its excellent therapeutic efficacy and improved target selectivity. Herein, a novel core-shell nanoparticle based on disulfide-crosslinked poly(methacrylic acid) (PMAA) layer coated polydopamine (PDA) particle has been successfully synthesized by precipitation polymerization. For these PDA@PMAA composite nanoparticles, PDA core exhibits high photothermal efficacy, meanwhile, the redox-labile PMAA shell serves as carriers to encapsulate anticancer drugs and selectively release them. Due to the characteristic of the disulfide bond, PMAA shell occurs at selective degradation as well as controlled drug release upon entering cancer cells. Moreover, the DOX-loaded PDA@PMAA nanoparticles demonstrated a synergistic effect, which shows a significantly improved inhibition effect against cancer cells by the combination of photothermal therapy and traditional chemotherapy with low drug dosage and short laser irradiation in an *in vitro* study.

**Electronic supplementary material:**

The online version of this article (10.1186/s11671-019-3027-6) contains supplementary material, which is available to authorized users.

## Introduction

Photothermal therapy (PTT), a non-invasive local cancer treatment, has been drawing great attention in cancer therapy for its high selectivity and minimal adverse effects [1]. In the PTT, the administered near-infrared (NIR) laser exposure, is absorbed by the photothermal conversion (PTC) agents and converted into local hyperthermia leading to tumor ablation [2–4]. A variety of nanomaterials have been revealed the PTC effect, such as gold nanostructures [5–7], carbon-based nanomaterials [8–12], Fe_3_O_4_ nanoclusters [13–15], CuS nanocrystals [16], and natural melanin [17], all of which exhibit strong optical absorbance in the NIR tissue optical window. Among these PTC agents, polydopamine (PDA), a mimic of the adhesive proteins found in mussels shows strong NIR absorption, high-PTC efficiency (40%), excellent biocompatibility, and biodegradability, which have been widely explored in the application of PTT [18, 19]. However, single use of PTT shows limited clinical efficacy due to insufficient heat delivery in target region without damaging surrounding normal tissues [20]. To address this problem, chemo-photothermal therapy with the combination of hyperthermia and chemotherapeutic agents has been exploited by many researchers for its synergistic effect resulted from the promoted drug delivery into tumors and increased drug toxicity by hyperthermia [21, 22].

To achieve optimized treatment effect, the current work is devoted to developing a novel therapeutic nanoparticle with high-performance photothermal conversion, excellent drug-loaded ability, and controlled drug release behavior. A “smart” polymer layer was introduced in our system, which crosslinked by a cleavable linker, to enable degradability and controlled drug release of carriers in a triggered fashion. Disulfide bond, which can be cleavage by free thiols, is a promising candidate as cleavable linker due to its sensitive response to redox state, high stability in blood circulation, and good biocompatibility [23]. Drug carriers incorporating disulfide bonds can undergo selective degradation upon entering tumor cells, in which the reducing glutathione (GSH) concentration (ca. 2–10 mM) is much higher than that in the extracellular fluids [24–26]. Herein, a new type of composite nanoparticles composed of PDA spheres as the core and disulfide-bond crosslinked poly(methacrylic acid) (PMAA) as the shell was prepared, denoted as PDA@PMAA, which maintains the PTC efficacy of PDA core and the redox-labile property of polymer shell. The structure, properties, and drug release behaviors of PDA@PMAA composite nanoparticles were studied, and chemo-photothermal therapeutic effect was further demonstrated via MTT assay.

## Methods/Experimental

### Materials

Dopamine hydrochloride (DA-HCl) and methacryloyl chloride and glutathione (GSH) were obtained from Aladdin Reagent Corporation, Shanghai, P.R. China. Methacrylic acid (MAA) and *N,N’*-bis(acryloyl)cystamine (BAC) was purchased from Sigma-Aldrich. 2,2-azobisisobutyronitrile (AIBN) was obtained from Sinopharm Chemical Reagent Company and recrystallized from ethanol. Ammonia aqueous solution (NH_3_•H_2_O, 30%), acetonitrile, and anhydrous ethanol were purchased from Shanghai Lingfeng Chemical Reagent Company. Doxorubicin (DOX) in the form of the hydrochloride salt was obtained from Beijing Huafeng United Technology Company. MTT (3-(4,5-dimethylthiazol-2-yl)-2,5-diphenyltetrazolium bromide) assay and other biological reagents were purchased from Invitrogen Corp. Calcein-AM was purchased from Bojin Biotech, Inc. (Xi’an). All chemical reagents were of analytical grade or better and used without further purification except as mentioned above.

### Characterization

Transmission electron microscopy (TEM) images were observed on a Tecnai G2 20 TWIN transmission electron microscope (FEI, USA). The hydrodynamic diameters and zeta potentials of particles were conducted by a dynamic light scattering (DLS) particle size analyzer (Malvern Nano-ZS90) at a scattering angle of 90°. UV-vis spectra were performed by a Perkin-Elmer Lambda 750 spectrophotometer at room temperature. Fourier-transform infrared (FT-IR) spectra were recorded using KBr-pressed plates on a Nicolet 6700 FTIR spectroscopy. The NIR-heating effects of PDA and PDA@PMAA nanoparticles were characterized using an 808-nm continuous-wave NIR laser (Changchun New Industries Optoelectronics Technology, Changchun, China; spot size: 6 mm × 7 mm) with laser irradiation at a power density of 5 W cm^−2^ for 300 s. Pre- and post-illumination temperatures were measured by a thermocouple with an accuracy of 0.1 ^°^C. The cellular images were acquired with a confocal laser scanning microscope (CLSM, Leica TCS SP8 STED 3X).

### PDA@PMAA Composite Nanoparticles

The synthesis of PDA spheres was carried out in a mixed solution of deionized water (90 mL), ethanol (40 mL), and NH_3_•H_2_O (3 mL), adding with 0.5 g dopamine hydrochloride. The solution was stirred at 30 ^°^C for 24 h, and the product was centrifuged and washed. The PMAA shell was synthesized by distillation-precipitation polymerization referred our previous work. MAA (100 mg), BAC (10 mg), and AIBN (3 mg) were dissolved in 25 mL acetonitrile, followed by adding 50 mg of as-prepared PDA sphere. Then, the mixture was heated to 100 ^°^C stirred for 2 h, and the product was centrifuged and washed. The mass ratio of PDA and MAA were varied from 0.5 to 6 to tune the thickness of PMAA shell, and details of the recipe were listed in Table 1.

**Table 1:** The recipes of PDA and PDA@PMAA composite nanoparticles

Sample	PDA (mg)	MAA (mg)	AIBN (mg)	BAC (mg)
PDA	/	/	/	/
PDA@PMAA-0.5^a^	50	25	0.75	2.50
PDA@PMAA-1	50	50	1.50	5.00
PDA@PMAA-2	50	100	3.00	10.00
PDA@PMAA-4	50	200	6.00	20.00
PDA@PMAA-6	50	300	9.00	30.00

^a^The number means the mass ratio of MAA to PDA spheres in the preparation

### Photothermal Effects of PDA@PMAA

The aqueous dispersion of PDA@PMAA (50 μg mL^−1^) placed in 96-well cell plate (100 μL per well) was illuminated by an 808-nm NIR laser (5 W cm^−2^), and pre- and post-illumination temperatures were measured.

### Drug Loading and Release

DOX was chosen as a model drug to investigate the drug loading and controlled release performance of PDA or PDA@PMAA nanoparticles. Specific steps referred to our previous work [27, 28]. Briefly, 10 mg of PDA or PDA@PMAA-1 nanoparticles were dispersed into 1 mL of DOX aqueous solution (1 mg mL^−1^), which was prior adjusted to pH8.0. After gentle stirring for 24 h at room temperature, the dispersion was centrifuged to collect the DOX-loaded PDA@PMAA nanoparticles (12,000 rpm, 10 min) and then washed with deionized water twice to remove unloaded DOX. UV absorbance spectra of supernatant were measured, and the intensity at 480 nm was used to analyze the loading and release of DOX. The drug loading content (LC) and encapsulation efficiency (EE) were expressed according to the following formulas: LC (%) = (weight of loaded drug)/(total weight of nanoparticles); EE (%) = (weight of loaded drug)/(weight of initially added drug).

In vitro release study was performed at 37 °C in phosphate buffer (pH7.4 and 5.5) with or without GSH. Typically, DOX-loaded PDA@PMAA nanoparticles dispersed in the corresponding buffer were placed into a dialysis bag (molecular weight cut-off 14,000 Da) and then immersed in 100 mL of the release medium. The samples were kept at 37 ^°^C under continuous shaking. At different time points, 2 mL of external buffer was taken out for UV-vis spectra analysis and replenished with an equal volume of fresh medium. All DOX release data were averaged over three measurements, and the releasing content was calculated by the formula: Releasing content (%) = (amount of drug in the release medium)/(amount of drug loaded into nanoparticles) × 100. The drug release behavior of DOX-loaded PDA@PMAA nanoparticles with laser irradiation in pH7.4 phosphate buffer was performed following a similar procedure. NIR light was applied for 5 min per hour.

### In Vitro Cell Assay

HEK-293T cells (human embryonic kidney cells, normal cells) and A549 cells (human lung adenocarcinoma epithelial cells, cancer cells) were cultured in Dulbecco’s modified Eagle’s medium (DMEM) supplemented with 10% FBS (fetal bovine serum), penicillin (100 U mL^−1^) and streptomycin (100 mg mL^−1^) in a humidified atmosphere with 5% CO_2_ at 37 °C.

To observe the cellular uptake, A549 cells (1 × 10^4^ cells per well) were seeded into 6-well plate in 1.5 mL of medium. The medium was replaced by the medium containing DOX-loaded PDA@PMAA nanoparticles after 24 h. After 1 h or 4 h, fresh DMEM and PBS were added to wash off the free nanoparticles not internalized by cells before fluorescence observation. The intracellular distribution of the nanoparticles was observed by CLSM. The fluorescence was imaged at *λ*_EX_ (488 nm) for DOX, and the false color was artificially set as red.

The cytotoxicity of DOX-loaded and blank PDA@PMAA nanoparticles against A549 cells were assessed by the standard MTT assay. The cells (with a density of 10^4^ cells per well) were incubated in 96-well plates for 24 h to allow cell attachment. Then, DOX-loaded and blank PDA@PMAA nanoparticles, and free DOX at various concentrations were added to the cells, respectively. For the NIR laser groups, the laser (*λ* = 808 nm) was applied to irradiate the cells at a power density of 5 W cm^−2^ for 300 s after 1 h incubation. Then, after an incubation time of 24 h at 37 ^°^C, 20 μL of MTT solution (5 mg mL^−1^ in phosphate buffer) was replaced with fresh DMEM containing MTT (5 mg mL^−1^), and the cells were incubated for another 4 h. Next, the supernatant was removed, and 150 μL of dimethyl sulfoxide (DMSO) was added to each well to dissolve the formazan. The absorbance was monitored at 570 nm using a spectrophotometer after 10 min of incubation. Each data point was collected by averaging that of five wells, and the untreated cells were used as controls. Percentage cell viability was calculated by comparing the absorbance of the control cells to that of treated cells. The same process of cytotoxicity of blank composite nanoparticles against HEK-293T cells was performed as above mentioned.

To visualize the antitumor effects of the PDA@PMAA nanoparticles and DOX-loaded PDA@PMAA nanoparticles with or without NIR laser irradiation, cells were seeded in 6-well plates at a density of 3 × 10^4^ cells per well. The cells were exposed to the PDA@PMAA-1 nanoparticles, DOX-loaded PDA@PMAA-1 nanoparticles, or free DOX for 24 h at a nanoparticles concentration of 100 μg mL^−1^ or equivalent DOX concentration of 5 μg mL^−1^. For the NIR laser groups, the laser (*λ* = 808 nm) was applied to irradiate the cells at a power density of 5 W cm^−2^ for 300 s after 1 h incubation. Subsequently, the cells were incubated with Calcein-AM for 30 min, washed three times using PBS, and observed using CLSM at *λ*_EX_ (490 nm).

## Results and Discussion

### Preparation and Characterization of PDA@PMAA Nanoparticles

**Fig. 1:**
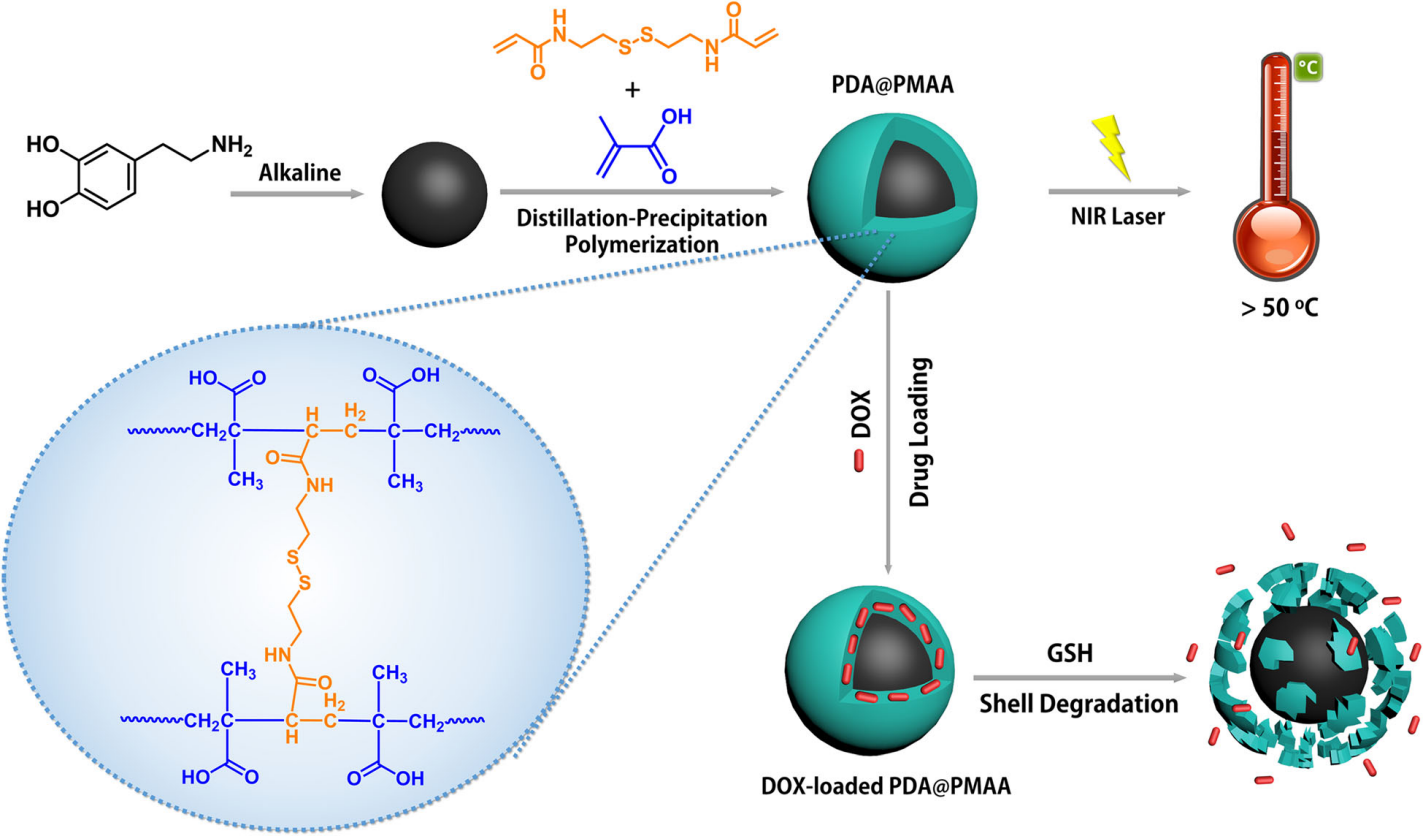
Schematic illustration of the synthesis, photothermal effect, drug loading, and stimuli-responsive drug release of PDA@PMAA nanoparticles

**Fig. 2:**
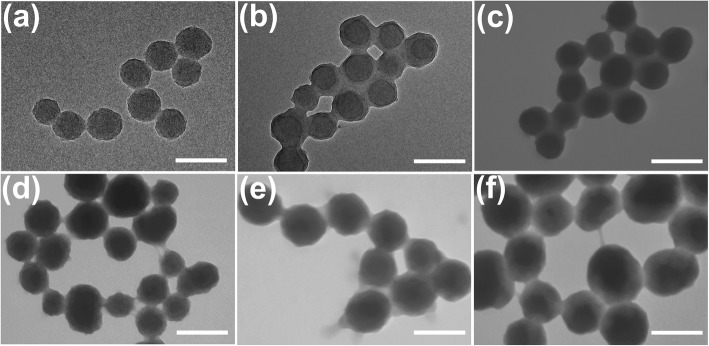
TEM images of PDA@PMAA nanoparticles (scale bar, 200 nm). **a** PDA. **b** PDA@PMAA-0.5. **c** PDA@PMAA-1. **d** PDA@PMAA-2. **e** PDA@PMAA-4. **f** PDA@PMAA-6

PDA sphere is prepared under basic condition *via* solution-oxidation method. The chemical coating of PDA sphere with a disulfide-crosslinked polymer layer was achieved through distillation-precipitation polymerization method that uses MAA and BAC as the monomer and crosslinker, respectively (Fig. 1). This multifunctional composite nanoparticle provides many advantages over other therapeutic nanoparticles in three aspects. First, the PDA core exhibits outstanding photothermal performance under NIR irradiation. Second, the incorporation of disulfide bond offers selective degradation of polymer shells as well as controlled drug release upon entering cancer cells. Third, the PMAA shell provides the nanoparticles with excellent colloidal stability. The thickness of PMAA layer could be controlled by adjusting the mass ratio of MAA and PDA spheres. Figure 2 shows the TEM images of the obtained PDA spheres and PDA@PMAA nanoparticles. It is clear that both PDA and PDA@PMAA nanoparticles are mono-dispersed and spherical in shape. The PDA spheres had an average diameter of ~ 100 nm, and the size of PDA@PMAA hybrid nanoparticles ranged from 120 ± 5 to 200 ± 10 nm with the mass ratio of PDA to MAA ranging from 0.5 to 6. The hydrodynamic size (D_h_) and size distribution of the PDA and PDA@PMAA nanoparticles were also characterized by dynamic light scattering (DLS), as shown in Table 2. The PDA@PMAA nanoparticles had a narrow size distribution, typically with a PI value of 0.09–0.14. The D_h_ of the series of PDA@PMAA nanoparticles were ranging from 176 to 349 nm by varying the mass ratio of PDA to MAA, which was in accordance with the trend of size growth from TEM observation. Notably, the D_h_ of composite nanoparticles were larger than the size determined by TEM, suggesting that the composite nanoparticles are highly swollen in aqueous medium [29]. The ζ potential of PDA nanoparticles was − 26.8 mV because of the catechol groups on the surface of PDA. The ζ potential of PDA@PMAA nanoparticles changed from − 30.2 to 33.2 indicating the existence of carboxyl groups comes from the PMAA shell.

**Table 2:** The colloidal data of PDA and PDA@PMAA composite nanoparticles

Sample	D_h_^a^ (nm)	PDI^b^	ζ potential^c^ (mV)
PDA	140	0.09	− 26.8
PDA@PMAA-0.5	176	0.16	− 30.2
PDA@PMAA-1	190	0.09	− 32.2
PDA@PMAA-2	227	0.14	− 31.7
PDA@PMAA-4	250	0.11	− 33.2
PDA@PMAA-6	349	0.24	− 33.0

^a^The hydrodynamic diameter (D_h_) was determined in PBS (pH7.4) at 25 ^°^C

^b^PDI, polydispersity index of the particle size

^c^ζ potential was determined in phosphate buffer (pH7.4) at 25 ^°^C

**Fig. 3:**
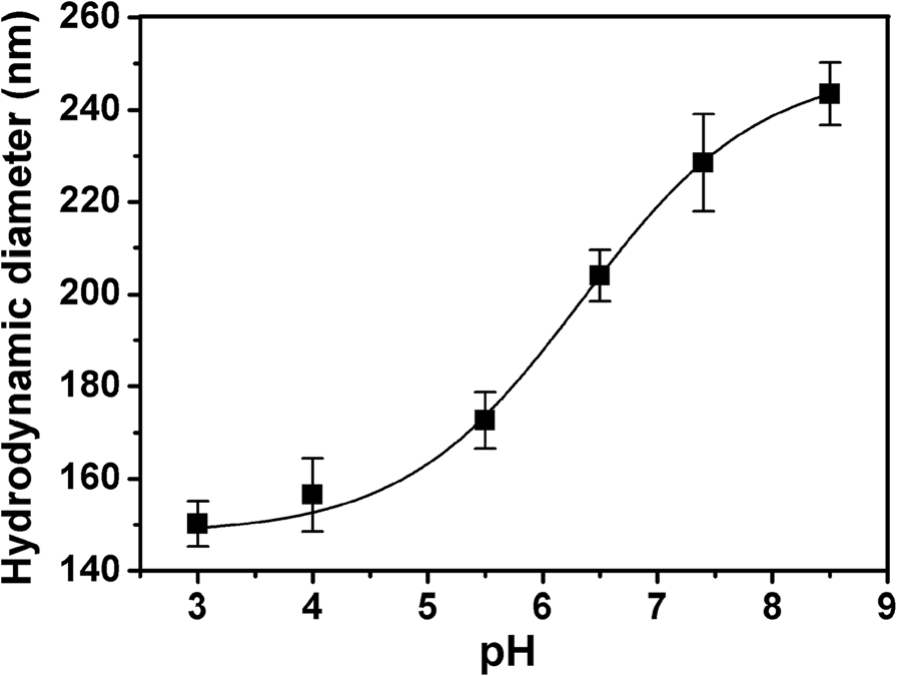
Hydrodynamic diameter of PDA@PMAA-1 nanoparticles in phosphate buffer at various pH

**Fig. 4:**
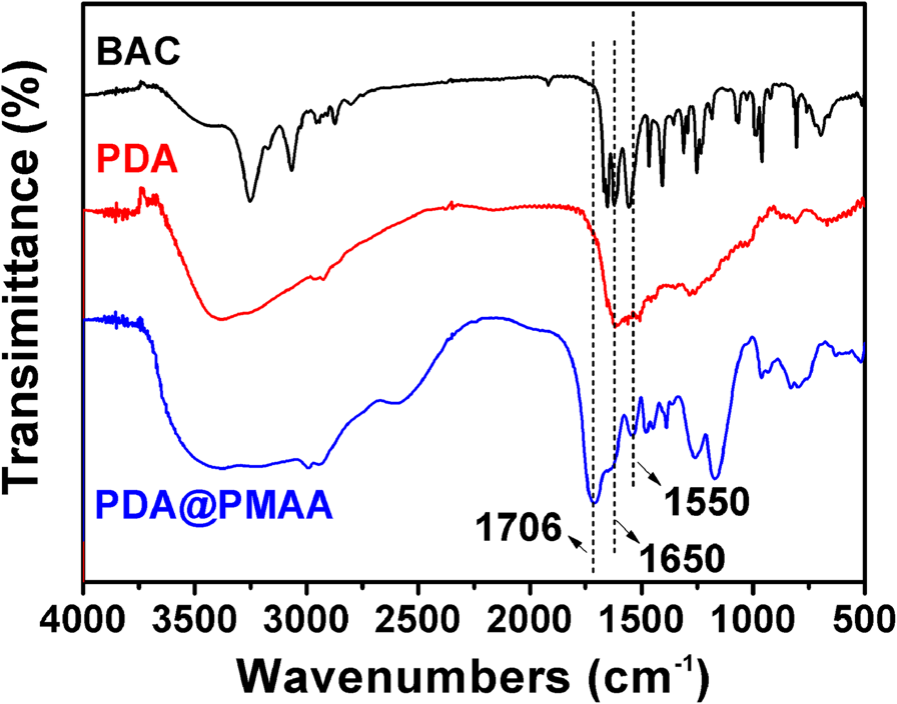
FTIR spectra of BAC crosslinkers, PDA nanoparticles, and PDA@PMAA nanoparticles (from top to bottom)

Since MAA is pH-responsive, it can be inferred that PDA@PMAA nanospheres also have a pH sensitivity. As shown in Fig. 3, the PDA@PMAA-1 nanoparticles were taken as an example to investigate the pH-dependence of the hydrodynamic size of PMAA coated nanoparticles. It can be seen that in phosphate buffer of pH8.5, the PDA@PMAA-1 nanoparticles have a hydrodynamic diameter of about 240 nm; while in an acidic environment of pH 3.0, their hydrodynamic size greatly shrank to ca. 150 nm. Because the PMAA polymer chains are highly ionized at high pH, and the strong electrostatic repulsion between polymer chains resulted in an enlargement of hydrodynamic size, while at low pH, the low ionization degree of PMAA chains leads to a size shrink [30]. The pH-responsive PDA@PMAA nanoparticles exhibit great potential in controlled drug release in tumor cells (pH lower than 6.5) since the collapse of the sponge-like polymer layer could facilitate drug release. The chemical structures of PDA@PMAA nanoparticles were characterized by Fourier-transform infrared (FTIR) spectroscopy (Fig. 4). In the spectra of BAC and PDA@PMAA nanoparticle, the bands appearing at 1650 and 1550 cm^−1^, which are attributed to the typical amide I and II bands of BAC, demonstrated that the crosslinker BAC had been successfully introduced into the composite nanoparticle [25]. The peak at 1706 cm^−1^, which belongs to the stretching vibration of C=O groups in PMAA, can be clearly seen in PDA@PMAA nanoparticle other than PDA nanoparticle, suggesting the successful coating of PMAA layer.

### Photothermal Effect of PDA@PMAA Nanoparticles

**Fig. 5:**
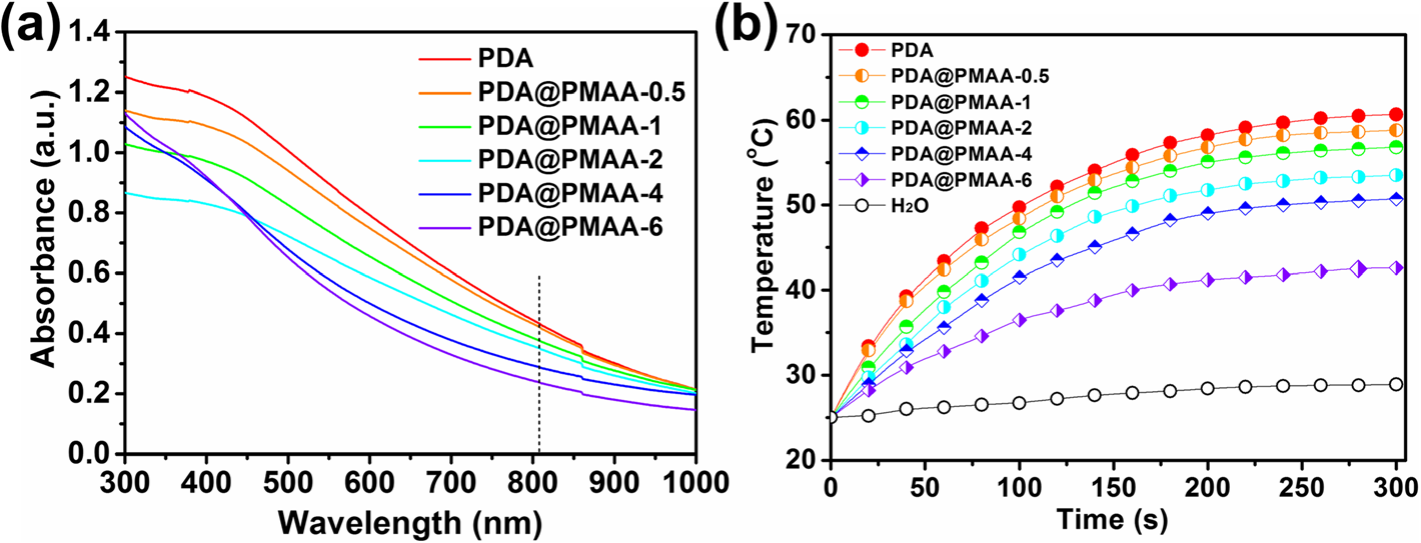
**a** UV-vis spectra of PDA and PDA@PMAA aqueous dispersion at a concentration of 100 μg mL^−1^. **b** Photothermal effect of PDA and PDA@PMAA aqueous dispersion at a concentration of 100 μg mL^−1^ were measured by laser irradiation (*λ* = 808 nm, 5 W cm^−2^)

The absorbance intensity in the NIR region is the main factor which determines the PTC capability of a PTC agent. To explore the light absorption ability of PDA@PMAA nanoparticles, their UV-vis spectra are summarized in Fig. 5a. It can be seen that each sample have an obvious absorption in the NIR region from 600 to 1000 nm. Compared with PDA@PMAA, PDA presents the highest absorption at 808 nm at an equivalent mass concentration. The absorbance of PDA@PMAA nanoparticles increased with the decrease in PMAA shell thickness (from PDA@PMAA-6 to PDA@PMAA-0.5). The strong optical absorption in the NIR region encouraged us to further investigate the photothermal effect of PDA@PMAA. As shown in Fig. 5b, the photothermal effect of PDA and PDA@PMAA aqueous dispersion were measured at the concentration of 100 μg mL^−1^ irradiated with an 808-nm laser at 5 W cm^−2^ for 300 s. Pure water was used as a negative control. The temperature of PDA dispersion was increased by 41 ^°^C and higher than all PDA@PMAA samples, which was consistent with its maximum absorption at 808 nm. The temperature increased by PDA@PMAA dispersions could reach from 17 to 33 ^°^C with the decrease of PMAA shell thickness (from PDA@PMAA-6 to PDA@PMAA-0.5), much higher than that caused by pure water control (only reach to 3.5 ^°^C). Previous studies suggested that thermal therapy with a temperature above 55 ^°^C showed great advantages in thermal ablation of solid tumor [31]. Comparing the maximum temperature rise of a series of PDA@PMAA nanoparticles, only PDA@PMAA-0.5 (58 ^°^C) and PDA@PMAA-1 (56 ^°^C) can reach up to 55 ^°^C. Considering the PMAA shell should have a certain thickness to ensure its drug loading capacity, PDA@PMAA-1 was chosen as representative in the following experiments.

### In Vitro Drug Release

**Fig. 6:**
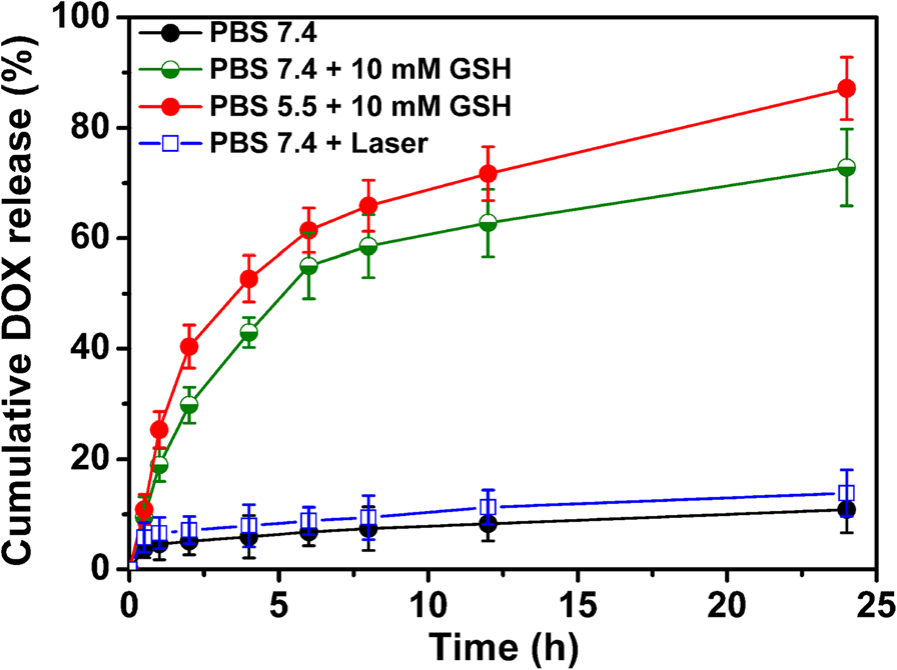
DOX release profiles of PDA@PMAA-1 nanoparticles at 37 ^°^C in 7.4 phosphate buffer (○), in 7.4 phosphate buffer with NIR laser irradiation (□, in 7.4 phosphate buffer with 10 mM GSH (red circle), or in 5.5 phosphate buffer with 10 mM GSH (green cirlce)

Doxorubicin (DOX) was chosen as a model drug to confirm the potential ability of PDA@PMAA-1 composite nanoparticles to release the encapsulated drug under reductive condition. Loading of DOX into composite nanoparticles was performed at a theoretical drug-loading content of 9.1 wt% and a polymer concentration of 10 mg mL^−1^, and the final drug-loading content and encapsulation efficiency was 5.1% and 53.7%, respectively. It indicated that DOX can be efficiently loaded into the polymer network. DOX-loaded PDA nanoparticles were also prepared for comparison, the DOX-loading content of which was 3.7%. The higher drug-loading capacity of PDA@PMAA-1 nanoparticles may be attributed to the strong electrostatic interaction between the amino group of the DOX molecules and the carboxyl groups of PMAA chains [25]. In view of that, the PMAA shell bears redox-cleavable disulfide bonds, the preloaded drugs will be triggered to efficiently release preloaded drugs in reducing conditions. Accounting for the slightly acidic tumor microenvironment and huge difference in GSH concentration between intracellular (1–10 mM) and plasma (20–40 μM), the in vitro drug release experiments were designed and conducted by using phosphate buffers of pH7.4 and 5.5 with or without 10 mM GSH to mimic the tumor cell and bloodstream environment [23, 32, 33]. As shown in Fig. 6, the release amount of drug is only 10.8% over a period time of 24 h, suggesting that DOX was held stably in the PDA@PMAA-1 nanoparticles when they were dispersed in physiological conditions. In the presence of 10 mM GSH, a remarkably quick release of drug was detected, where the cumulative release of DOX was approximately 72.8% within 24 h, due to the degradation of disulfide-bonded PMAA shells in reducing environment. Yet, the structure of PDA core maintains integrity after the redox-responsive degradation (Additional file 1: Figure S2). Moreover, a burst release (ca. 87% within 24 h) of DOX was observed in phosphate buffers of pH5.5 with 10 mM GSH, due to the carboxyl groups in PMAA being protonated under acidic conditions, which further caused collapsing of the polymer networks. These release profiles implied the promising feature of PDA@PMAA nanoparticles for controlled drug release as the nanoparticles exhibiting low leakage of drugs in plasma, however, releasing drugs fast when entering the tumor cells. Furthermore, a slow release of the drug (ca. 13% within 24 h) for DOX-loaded PDA@PMAA nanoparticles with NIR irradiation in phosphate buffers of pH7.4 was detected, indicating the PDA@PMAA nanoparticles maintaining structural integrity upon irradiation. The release behavior of PDA nanoparticles in the presence of 10 mM GSH showed a remarkable low release of drugs (ca. 30% in 24 h) in comparison with PDA@PMAA-1 nanoparticles (Additional file 1: Figure S1). The huge difference in release behaviors of PDA and PDA@PMAA nanoparticles suggested that the introduction of degradable polymer shell crosslinked by disulfide bond gave rise to the Redox-triggered effective release of drugs.

### Cell Assays

**Fig. 7:**
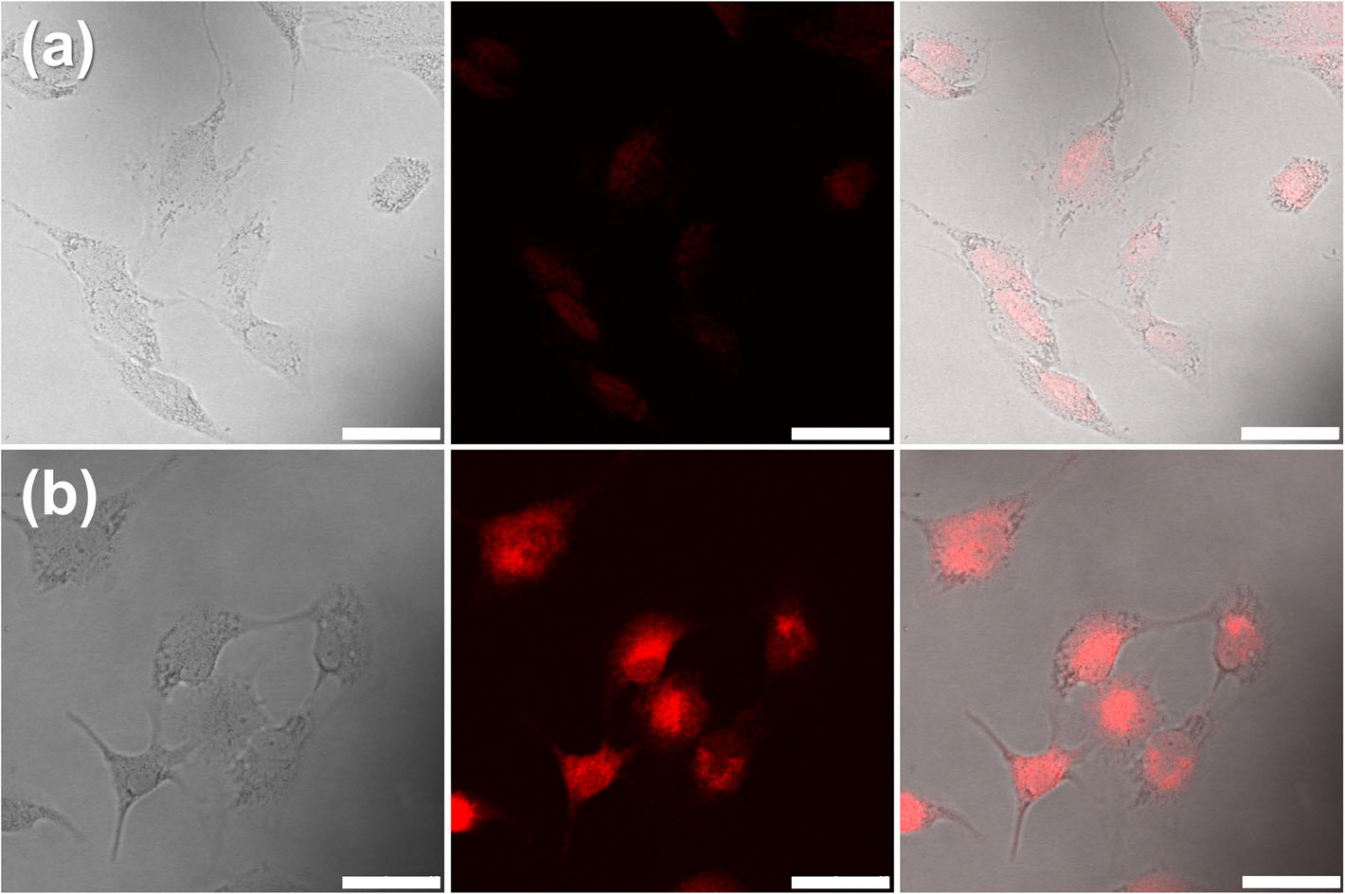
CLSM images of A549 cells cultivated with DOX-loaded PDA@PMAA nanoparticles for (**a**) 1 h and (**b**) 4 h. In each row, differential interference contrast microscopy images, fluorescence images, and overlay images were shown from left to right, respectively. (scale bar, 50 μm)

Investigation of the cellular uptake and intracellular drug release of DOX-loaded PDA@PMAA nanoparticles was further conducted against the A549 cell line. As shown in Fig. 7, red fluorescence for DOX can be observed in the cell cytoplasm after 1 h incubation, indicating a fast internalization of nanoparticles against tumor cells. After 4 h incubation, strong red fluorescence was observed throughout the cell cytoplasm and nucleus. It suggested that more nanoparticles were endocytized by cells and efficiently released DOX through the degradation of polymer shells in the reducing environment of tumor cells.

**Fig. 8:**
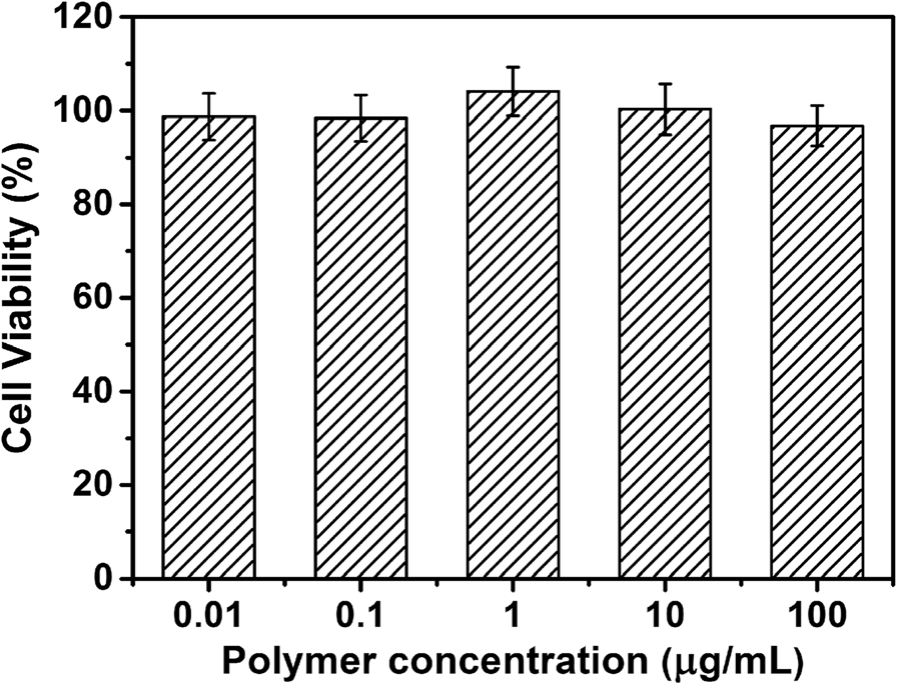
Cell viability of HEK-293 cells exposed to blank PDA@PMAA-1 nanoparticles for 24 h

**Fig. 9:**
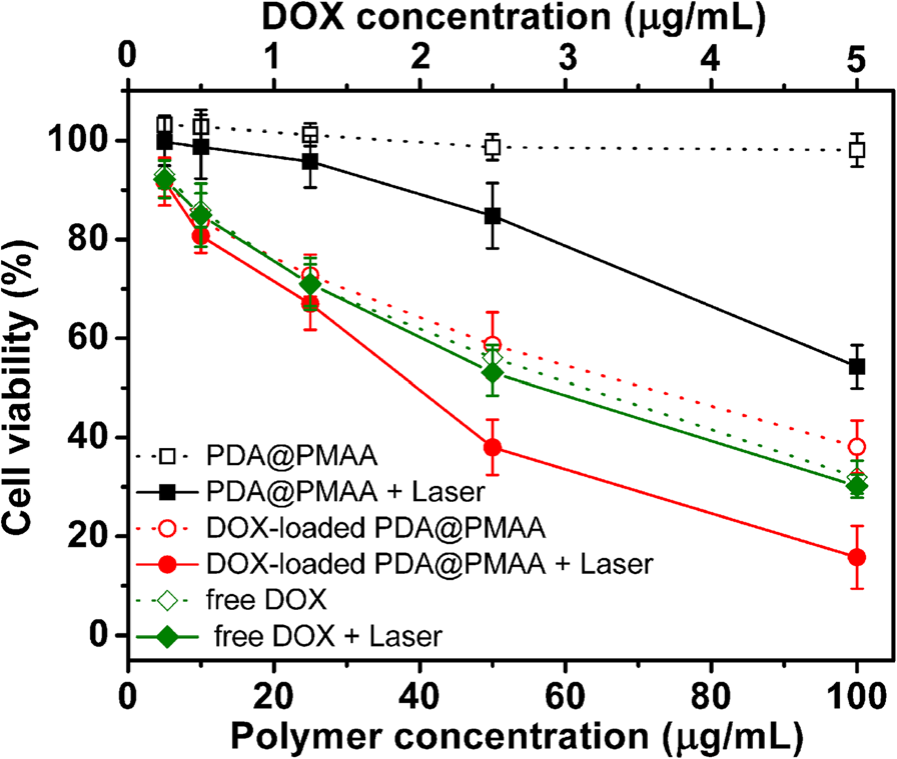
Cell viability of A549 cells treated with free DOX, PDA@PMAA-1 nanoparticles, and DOX-loaded PDA@PMAA-1 nanoparticles at various concentration without or with NIR laser irradiation (*λ* = 808 nm, 5 W cm^−2^) for 300 s

**Fig. 10:**
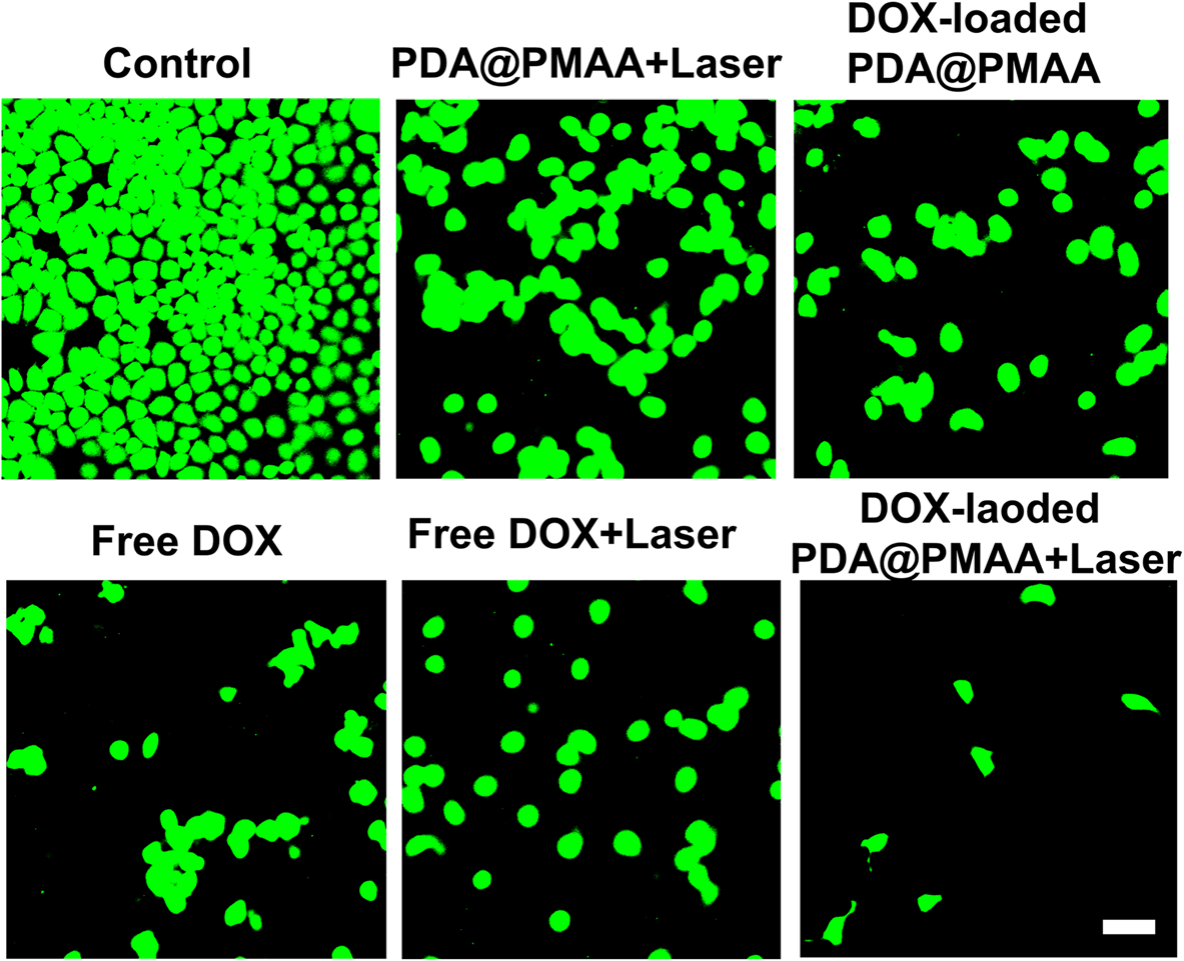
Confocal fluorescence images of live A549 cells treated with PBS, PDA@PMAA-1 nanoparticles, DOX-loaded PDA@PMAA-1 nanoparticles, and free DOX with or without NIR laser irradiation (*λ* = 808 nm, 5 W cm^−2^) for 300 s. Live cells were stained with Calcein-AM (green). The scale bar is 50 μm

To evaluate the biocompatibility of PDA@PMAA, a typical normal cell (HEK-293T cells) was chosen for the cell viability test by MTT assay. As shown in Fig. 8, no obvious cytotoxicity of blank PDA@PMAA-1 nanoparticles were detected at a wide concentration range of 0.1–100 μg mL^−1^, indicating the good biocompatibility of PDA@PMAA-1 nanoparticles which ensures their application in the biomedical area. Next, the cell viability against A549 cells (tumor cells) as a function of incubation concentration of blank or DOX-loaded PDA@PMAA-1 nanoparticles was measured, and each group was subdivided into groups with or without NIR laser exposure (Fig. 9). Almost no effect on cell viability was observed for the blank PDA@PMAA-1 group without laser, indicating that the blank composite nanoparticles have no cytotoxicity. After 5 W cm^−2^ NIR laser irradiation for 300 s, the cell viability for blank PDA@PMAA-1 group decreased obviously, and ~ 54.3% of cells were killed at a concentration of 100 μg mL^−1^. The results implied these PDA@PMAA-1 nanoparticles were cytotoxic against A549 cells by NIR irradiation inducing hyperthermia. As for DOX-loaded groups, DOX-loaded PDA@PMAA-1 nanoparticles shows a decrease of cell viability in a dose-responsive manner, which have similar efficacy to the free DOX, indicating a complete release of drugs from disulfide-bond crosslinked PMAA shells. For cells treated by DOX-loaded PDA@PMAA-1 nanoparticles with NIR laser exposure, the cell viability shows a deeper decrease compared with the non-irradiation group, especially at a high-drug dose. For example, when the cells were treated with 100 μg mL^−1^ DOX-loaded PDA@PMAA-1 (containing 5 μg mL^−1^ DOX), the cell viability was reduced to about 15.7%, which was much lower than photothermal (~ 54.3%) or chemotherapy (~ 38.1%) treatment alone under the same dose of nanoparticles. Notably, the 50% cell inhibition (IC_50_) value of DOX-loaded PDA@PMAA-1 with a short-term NIR laser exposure was determined to 2 μg mL^−1^, which was much lower than that of free DOX (6.3 μg mL^−1^). It suggests that the chemo-photothermal therapy of DOX-loaded PDA@PMAA nanoparticles demonstrated a synergistic effect, which may be attributed to the enhanced cytotoxicity of DOX at higher temperature [34, 35]. In contrast, the free DOX with NIR laser group does not show a similar synergistic effect since there is no local hyperthermia caused by NIR laser irradiation. Fluorescence images of Calcein-AM (green, live cells)-stained cells after treatment show that the number of live cells treated by DOX-loaded PDA@PMAA nanoparticles upon NIR laser irradiation was significantly less than other groups, which further confirmed the synergistic antitumor effect of DOX-loaded PDA@PMAA nanoparticles with NIR light irradiation (Fig. 10). Benefiting from the positive synergistic effect of chemo-photothermal combined treatment, it allows lower cytotoxic drug dosage to achieve the same tumor-killing effect, therefore avoiding the severe side effects to normal tissues at high-drug dosage. Taken together, the above data suggest these PDA@PMAA nanoparticles can efficiently release drugs under the intracellular reducing conditions and display a synergistic tumor cell killing effect for combined chemo-photothermal therapy, which demonstrated their great potential for cancer treatment.

## Conclusion

The multifunctional core-shell PDA@PMAA composite nanoparticles were synthesized by coating disulfide-crosslinked PMAA layer on PDA nanoparticles via distillation-precipitation polymerization. The composite nanoparticles exhibited an excellent photothermal conversion effect and redox-labile degradation of PMAA layer. For a typical sample PDA@PDA@PMAA-1, the temperature of PDA@PMAA dispersions was increased by 31 ^°^C at the concentration of 100 μg mL^−1^ irradiated with an 808 nm laser at 5 W cm^−2^ for 300 s. The DOX-loaded PDA@PMAA-1 nanoparticles were stable under the physiological environment with low leakage of DOX (10.8% in 24 h), while a rapid and full release of DOX was triggered in the reducing and weakening acidic condition (pH5.5 + 10 mM GSH). The cell viability of A549 cells treated with PDA@PMAA-1 nanoparticles under NIR irradiation was reduced significantly to about 15.7% at relatively low equivalent drug concentration (5 μg mL^−1^), which was much lower than photothermal (~ 54.3%) or chemotherapy (~ 38.1%) treatment alone under the same dose of nanoparticles and drugs. So the DOX-loaded composite nanoparticles realized a synergistic inhibition effect against cancer cells by the combination of photothermal therapy and traditional chemotherapy. This work demonstrated the feasibility of such composite nanoparticles to be a powerful platform for controlled drug delivery and could be exploited as combined chemo-photothermal therapy with improved therapeutic efficacy.

## Additional file

Additional file 1: **Figure S1.** DOX release profiles of PDA-DOX nanoparticles at 37 ^°^C in 7.4 phosphate buffer (□) or in 7.4 phosphate buffer with 10 mM GSH (■). **Figure S2.** TEM images of PDA@PMAA-1 nanoparticles degraded in 7.4 phosphate buffer with 10 mM GSH for 12 h. (scale bar, 200 nm). (DOCX 403 kb)

## Data Availability

The datasets generated during and/or analyzed during the current study are available from the corresponding author on request.

## Abbreviations

AIBN: 2,2-Azobisisobutyronitrile
BAC: *N,N'*-bis(acryloyl)cystamine
DA-HCl: Dopamine hydrochloride
DLS: Dynamic light scattering
DMEM: Dulbecco’s modified Eagle’s medium
DMSO: Dimethyl sulfoxide
DOX: Doxorubicin
FBS: Fetal bovine serum
FT-IR: Fourier-transform infrared
GSH: Glutathione
MAA: Methacrylic acid
NIR: Near-infrared
PDA: Polydopamine
PMAA: Poly(methacrylic acid)
PTC: Photothermal conversion agents
PTT: Photothermal therapy
TEM: Transmission electron microscopy
