# Supplementary material for: Polydopamine-Based Composite Nanoparticles with Redox-Labile Polymer Shells for Controlled Drug Release and Enhanced Chemo-Photothermal Therapy
Source: Nanoscale Res Lett. 2019 May 30;14:186. doi: 10.1186/s11671-019-3027-6 (PMC6542907; doi:10.1186/s11671-019-3027-6)
Supplement: Supplementary file 1 — Figure S1. DOX release profiles of PDA-DOX nanoparticles at 37 °C in 7.4 phosphate buffer (□) or in 7.4 phosphate buffer with 10 mM GSH (■). Figure S2. TEM images of PDA@PMAA-1 nanoparticles degraded in 7.4 phosphate buffer with 10 mM GSH for 12 h. (scale bar, 200 nm). (DOCX 403 kb) [file 11671_2019_3027_MOESM1_ESM.docx]

**Additional file 1**

**Polydopamine-based composite nanoparticles with redox-labile polymer shells for controlled drug release and enhanced chemo-photothermal therapy**

Yefei Tian^1,2^*, Miao Lei^1^

^1^ School of Materials Science and Engineering, Chang'an University, Xi'an 710064, Shaanxi, PR China

^2^ Engineering Research Central of Pavement Materials, Ministry of Education of PR China, Chang'an University, Xi'an 710064, PR China

* Corresponding Author: E-mail: yftian@chd.edu.cn; Tel 86 29 8233 7258

1. Supplementary figures





**Figure S1.** DOX release profiles of PDA-DOX nanoparticles at 37 ^o^C in 7.4 phosphate buffer (□) or in 7.4 phosphate buffer with 10 mM GSH (■).


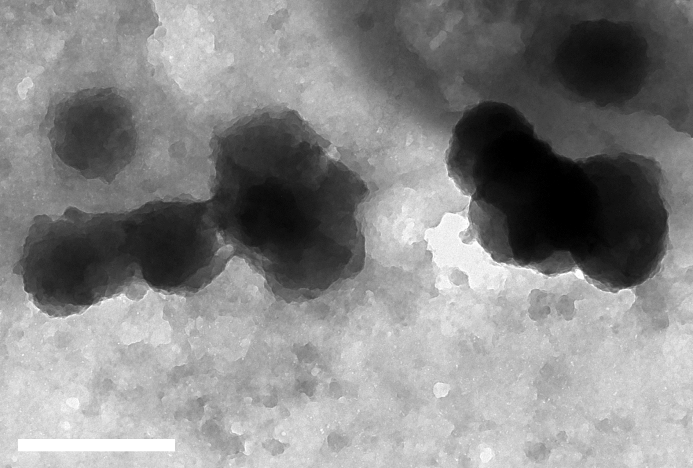


**Figure S2**. TEM images of PDA@PMAA-1 nanoparticles degraded in 7.4 phosphate buffer with 10 mM GSH for 12 h. (scale bar, 200 nm)
